# A biofertilizing fungal endophyte of cranberry plants suppresses the plant pathogen *Diaporthe*

**DOI:** 10.3389/fmicb.2024.1327392

**Published:** 2024-02-02

**Authors:** Bhagya C. Thimmappa, Lila Naouelle Salhi, Lise Forget, Matt Sarrasin, Peniel Bustamante Villalobos, Bernard Henrissat, B. Franz Lang, Gertraud Burger

**Affiliations:** ^1^Department of Biochemistry, Robert-Cedergren Centre for Bioinformatics and Genomics, Université de Montréal, Montreal, QC, Canada; ^2^DTU Bioengineering, Technical University of Denmark, Lyngby, Denmark; ^3^Department of Biological Sciences, King Abdulaziz University, Jeddah, Saudi Arabia

**Keywords:** alternative splicing, biocontrol, *Codinaeella* sp., *Diaporthe vaccinii*, hydrolytic enzymes, secondary metabolites, sustainable agriculture, *Vaccinium macrocarpon* Aiton (American cranberry)

## Abstract

Fungi colonizing plants are gaining attention because of their ability to promote plant growth and suppress pathogens. While most studies focus on endosymbionts from grasses and legumes, the large and diverse group of ericaceous plants has been much neglected. We recently described one of the very few fungal endophytes promoting the growth of the Ericaceae *Vaccinium macrocarpon* (American cranberry), notably the *Codinaeella* isolate EC4. Here, we show that EC4 also suppresses fungal pathogens, which makes it a promising endophyte for sustainable cranberry cultivation. By dual-culture assays on agar plates, we tested the potential growth suppression (or biocontrol) of EC4 on other microbes, notably 12 pathogenic fungi and one oomycete reported to infect not only cranberry but also blueberry, strawberry, tomato plants, rose bushes and olive trees. Under greenhouse conditions, EC4 protects cranberry plantlets infected with one of the most notorious cranberry-plant pathogens, *Diaporthe vaccinii*, known to cause upright dieback and berry rot. The nuclear genome sequence of EC4 revealed a large arsenal of genes potentially involved in biocontrol. About ∼60 distinct clusters of genes are homologs of secondary metabolite gene clusters, some of which were shown in other fungi to synthesize nonribosomal peptides and polyketides, but in most cases, the exact compounds these clusters may produce are unknown. The EC4 genome also encodes numerous homologs of hydrolytic enzymes known to degrade fungal cell walls. About half of the nearly 250 distinct glucanases and chitinases are likely involved in biocontrol because they are predicted to be secreted outside the cell. Transcriptome analysis shows that the expression of about a quarter of the predicted secondary-metabolite gene clusters and glucan and chitin-degrading genes of EC4 is stimulated when it is co-cultured with *D. vaccinii*. Some of the differentially expressed EC4 genes are alternatively spliced exclusively in the presence of the pathogen, altering the proteins’ domain content and subcellular localization signal, thus adding a second level of proteome adaptation in response to habitat competition. To our knowledge, this is the first report of *Diaporthe*-induced alternative splicing of biocontrol genes.

## 1 Introduction

Essentially all plants are associated with microbes, either internally colonizing different plant parts or externally present on the surface. Among the microbes residing inside the plant (referred to as endophytes), some engage in a mutualistic relationship with their host by improving the plant’s nutrient uptake (biofertilization) and inhibiting plant pathogens (biocontrol), while others are plant pathogens themselves.

Microorganisms associated with plants have been studied for decades with a focus on grass (Poaceae) and legume (Fabaceae) symbionts (Harrison and Griffin, 2020; Pirttilä et al., 2021), but only little is known about endophytes colonizing the large and diverse group of ericaceous plants. The Ericaceae are one of the most species-rich families of flowering plants, containing 4,250 known species from 124 genera. They are commonly known as the heather family and are often found in nutrient-poor and acidic soil (Stevens et al., 2004). Unexpectedly, Ericaceae do not host the well-known Arbuscular Mycorrhizal fungi (Glomeromycota), which otherwise colonize more than 90% of all land-plant species. Instead, most fungi isolated from Ericaceae belong to the Ascomycota, including Helotiales, Chaetothyriales, and Xylariaceae (Allen et al., 2003; Bougoure and Cairney, 2005; Zhang et al., 2016; Yang et al., 2018), and a few are Basidiomycota from the order Sebacinales (Berch et al., 2002; Allen et al., 2003). *Vaccinium macrocarpon* (cranberry) is one of the economically most important Ericaceae crop plants. Particularly in milder climates, cranberry is prone to diseases (Hisano et al., 2012; University of Massachusetts Cranberry Station, 2016), which may explain why most of the published work on cranberry-associated microbes bears on pathogens. Only a few reports have focused on the beneficial effects of cranberry symbionts, such as the stimulation of nitrogen influx into the plant (Kosola et al., 2007) and pathogen suppression (Mason et al., 2014).

Recently, we isolated and characterized a large number of bacterial and fungal endosymbionts of cranberry plants and measured their biofertilizing and biocontrol ability (Salhi et al., 2022). The *Codinaeella* sp. isolate EC4 proved to promote cranberry-plant growth (Thimmappa et al., 2023) and, in addition, to inhibit the growth of two cranberry-plant pathogens, *Godronia* sp. and *Cytospora chrysosperma* (Salhi et al., 2022). Here, we aim to unravel the biocontrol mechanism of EC4. To achieve this, we investigated the effect of EC4 on a broader range of plant pathogens, searched for homologs of known biocontrol genes in EC4’s nuclear genome (Thimmappa et al., 2023) and examined their transcriptional expression. We observed that the fungal plant pathogen *Diaporthe vaccinii* triggers differential expression of EC4 genes tentatively involved in biocontrol and promotes alternative splicing of a subset of these genes. These phenomena indicate that much of the biocontrol activity of EC4 is induced upon the encounter with the pathogen.

## 2 Materials and methods

### 2.1 Strains and culture conditions

The ten presumed pathogenic fungal strains (with isolate designation given in parenthesis) were isolated from the following environments: *Physalospora vaccinii* (C1), *Colletotrichum gloeosporioides* (EC77), and *Godronia cassandrae* (EC82) from the surface of diseased cranberries; *Rhizopus* sp. (F1), *Cadophora luteo-olivacea* (F4) and *Botrytis cinerea* (F5) from the surface of diseased strawberries; *Alternaria alternata* (B1) from the surface of diseased blueberries; and *Alternaria alternata* (T1 and T2) from the surface of diseased tomatoes. All fruit were grown in Quebec and purchased from Montreal grocery stores. *Botrytis cinerea* (R1) was isolated from diseased Quebec-grown rose flowers (Supplementary Tables 1, 2).

*Alternaria alternata* (IS2), *Peniophora* sp. (IS5), *Diaporthe vaccinii* (IS7) and *Penicillium* sp. (IS8) were isolated by Dr. Richard Belanger’s group at Laval University from diseased Quebec-grown cranberries. *Verticillium dahliae* VD1, *Verticillium dahliae* VD2, *Fusarium graminearum* FG5, *Fusarium graminearum* FG6, *Phytophthora infestans* PI3, and *Phytophthora infestans* PI4 were isolated from olive trees and kindly provided by Dr. Konstantinos A. Aliferis, Agricultural University of Athens, Greece. Most of the fungal strains mentioned here grew readily on potato dextrose agar (PDA) and yeast glycerol growth medium except for *Verticillium* (VD1 and VD2) and *Phytophthora* (PI3 and PI4), which require a V8 agar medium (Supplementary Tables 1, 2).

### 2.2 DNA extraction and fungal identification

All the fungal strains were grown in a glycerol yeast extract liquid medium (2% glycerol, 0.4% yeast extract, pH 7). Mycelia were mechanically broken using a mortar and pestle with glass beads (425–600 μm, Sigma). DNA was then extracted using a Qiagen Genomic-tips 20/G (Qiagen, GmbH) following the manufacturer’s recommendation. Fungal ribosomal internal transcribed spacer regions were amplified using BMBC-F and ITS4-R primers as described elsewhere (Thimmappa et al., 2023), and the PCR products were purified using Wizard^®^ SV Gel and PCR Clean-Up System (Promega, USA). Amplicons were sequenced, and reads were assembled using PHRAP v1.090518 (de la Bastide and McCombie, 2007) and visualized with Consed v27.0 (Gordon et al., 1998). Species were identified by BLASTN^1^ searches against the NCBI non-redundant (nr) database. Multiple matches with the same taxon among the ten best hits were considered correctly identified. Matches with more than one taxon among the top ten were labeled ambiguous.

### 2.3 Preparation of fungal inoculum

Fungi were grown in a potato dextrose broth at room temperature for 4 days. The mycelia were filtered and ground in a blender for 3 min (Hamilton Beach, model 51109C, 225W). After serial dilution, the titer was plated on a potato dextrose agar plate, and colony forming units (CFUs) were counted. The titer was adjusted to 1 × w^6^ CFU/ml for inoculation.

### 2.4 Screening of growth suppression (biocontrol) activity on agar plates

Biocontrol analysis was performed by placing 5 mm of 5-day-old mycelia of EC4 and pathogens on an agar plate at a 30 mm distance. After incubation for 15 days at 25°C, the growth radius of the pathogen was measured. The percentage of radial growth inhibition (PRI) was calculated using the equation: PRI = (r1-r2/r1) × 100, where r1 is the radial colony growth on the control plate, and r2 is the radial colony growth in the respective screening experiment. To test if residual pathogen mycelium was present in the inhibition zone, the dual-culture plate was inspected under a Nikon SMZ stereo microscope. A petri dish colonized by *Diaporthe* was inoculated with an agar block of 5 mm^2^ of 15 days-old EC4 inoculum. After 3 weeks, the top and bottom view of the clearance zone was recorded.

### 2.5 *In planta* pathogenicity test

Cranberry plantlets were raised aseptically from seeds, as described elsewhere (Thimmappa et al., 2023). In brief, surface-sterilized cranberry seeds were germinated in a culture box containing gelzan, sucrose, NaFe, MgSO_4_, KNO_3_, KCL, KH_2_PO_4_, Ca(NO_3_), KI, MnSO_4_, ZnSO_4_, CuSO_4_, and vitamins (for more detailed information on the growth medium, see Thimmappa et al., 2023) and grown under 16 h light and 8 h dark conditions at room temperature. After 3 months, two to three leaves of each plant were inoculated with presumed fungal pathogens (see Section “2 Materials and methods” and Supplementary Table 1). The *in planta* test was performed in three cultivation boxes, each holding nine plants. Plants were regularly inspected visually for any disease development.

### 2.6 *In planta* biocontrol test

Two to three leaves of each of 27 aseptically grown three-month-old cranberry plantlets (again three boxes, each holding nine plants) were inoculated with 10 CFU/leaf with either EC4 alone, *Diaporthe* alone or EC4 and *Diaporthe* together. Cranberry plants were inoculated with 3 μl of sterile distilled water as a control. Plants were visually inspected after 10, 40, 70 and 120 days.

### 2.7 Test for secreted antimicrobial compounds

A liquid yeast glycerol medium in which either *Diaporthe* or EC4 was grown alone, or EC4 was grown together with *Diaporthe* for 1 to 4 days was tested for antimicrobial compounds. The medium was concentrated 20× using a speed vac at 4°C and then filtered (0.22 μm pore size). *Diaporthe* was inoculated at the center of the culture plate, and the 50 μl of filtrates were placed 3 cm from the center, with yeast glycerol medium as a control. Pathogen growth at room temperature was monitored for 15 days.

### 2.8 Co-culture of fungi and RNA isolation

To test RNA expression of EC4 in the presence of *Diaporthe*, ∼1 × 10^6^ CFUs of EC4 and the pathogen were inoculated into 50 mL of glycerol yeast extract liquid medium. The control experiments with EC4 grown alone are reported in our previous work (Thimmappa et al., 2023). In both experiments, cultures were grown under shaking at room temperature for 3 days. The RNA was isolated with the RNeasy Plus Universal kit from Qiagen following the manufacturer’s recommendations.

### 2.9 RNA-Seq library construction, sequencing, and differential gene expression analysis

The construction of stranded poly-A RNA libraries and Illumina paired-end sequencing (100-bp read length) were outsourced to the Genome Quebec Innovation Center in Montreal. Adapter sequences were trimmed using Trimmomatic v0.35 (Bolger et al., 2014) and reads aligned to the genome using STAR v2.7.1 with –quantMode TranscriptomeSAM parameter (Dobin et al., 2013). Gene and isoform expression were estimated using RSEM v1.3.3 (Li and Dewey, 2011). Differential gene expression analysis was performed with three biological replicates using the DESeq2 (Love et al., 2021) tool. Genes were considered up and down-regulated if the log2 fold change was ≥ +1 and ≤ −1, respectively, and using a false-discovery rate cutoff of 0.05. A biosynthetic gene cluster was considered differentially upregulated when the core-biosynthetic gene was differentially upregulated. RNA-Seq data sets are deposited under the NCBI BioProject ID: PRJNA831867.

### 2.10 Statistical data analysis

For the statistical analysis of growth inhibition, the Kruskal–Wallis rank-sum test was performed using the package FSA (Ogle et al., 2023), followed by the Dunn or pairwise Mann-Whitney test as a multiple comparison *post hoc* analysis. Differences with *p*-values of < 0.05 were considered statistically significant.

### 2.11 Prediction of fungal cell wall degrading enzymes

CAZyme families were identified in the inferred EC4 proteome using the methodology that is applied for the regular updates of the CAZy database,^2^ along with expert validation (Cantarel et al., 2009; Lombard et al., 2014).

### 2.12 Prediction of biosynthetic gene clusters

Secondary metabolite biosynthetic gene clusters (BGCs) were identified genome-wide using antiSMASH (antibiotics and Secondary Metabolite Analysis) v.7.0.0 for fungi (Blin et al., 2023), with the following options: detection strictness: strict, KnownClusterBlast, ClusterBlast, SubClusterBlast, minimum information about a biosynthetic gene (MIBiG) cluster comparison, ActiveSiteFinder, RREFinder, Cluster Pfam analysis, Pfam-based GO term annotation and TIGRFam analysis.

### 2.13 Secretome prediction

The prediction of secreted proteins in the inferred EC4 proteome proceeded in several steps. First, proteins carrying an N-terminal signal peptide were predicted cumulatively using SignalP v5.0 (Bendtsen et al., 2004), Phobius v1.01 (Käll et al., 2004) and TargetP v2.0 (Almagro Armenteros et al., 2019) with default parameters. Then, proteins that potentially attach to membranes or carry signals for retention in certain subcellular locations were identified by TMHMM v2.0 (Krogh et al., 2001) and Phobius v1.01 (Käll et al., 2004) and tested for the presence of a Glycosylphosphatidylinositol(GPI)-anchor predicted by the PredGPI tool (Pierleoni et al., 2008), and the Prosite motif “PS00014” predicted by PS-Scan (de Castro et al., 2006). These proteins were removed from the set carrying a signal peptide determined in Step 1.

### 2.14 Alternative splicing events

Genome-wide Alternative Splicing (AS) events recorded in the annotation (.gtf) file were classified using SUPPA2 (Trincado et al., 2018). Secretory peptides were predicted as described above. Protein domains were predicted using Pfam-scan.pl v1.6 (Finn et al., 2010). Domains were analyzed manually to pinpoint the consequences of AS events. Graphs were generated using IsoformSwitchAnalyzeR v2.1.2 (Vitting-Seerup and Sandelin, 2019).

## 3 Results and discussion

### 3.1 EC4 inhibits the growth of a broad range of plant pathogens

*Codinaeella* sp. isolate EC4 is a phytostimulating fungal endosymbiont of cranberry, which was shown in *on-plate* assays to inhibit the growth of two fungal pathogens, *Cytospora chrysosperma* and *Godronia cassandrae* (Salhi et al., 2022; Thimmappa et al., 2023). Here, we examined which other pathogens are suppressed by EC4 and validated their pathogenicity in cranberry plants.

To test the biocontrol ability of EC4, the endophyte was inoculated on an agar plate together with several presumed plant pathogens (for details, see “2 Materials and methods”). These microbes, mostly Ascomycota, Basidiomycota, and Mucoromycota, have been reported to infect not only cranberry plants but also blueberry, strawberry, tomato, rose plants and olive trees. A strain of the oomycete *Phytophthora infestans* isolated from olive trees was also tested (Supplementary Table 1).

**FIGURE 1:**
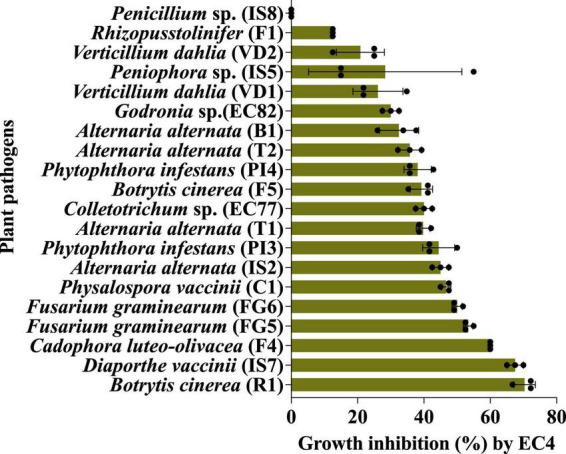
The *in vitro* biocontrol activity of EC4 against a broad range of other fungi and oomycetes was measured by a confrontation test on agar plates. The statistical support of growth inhibition was *P* < 0.05, calculated by the Kruskal–Wallis test (Ogle et al., 2023) (for details, see “Materials and methods”). All the tests were done in triplicates and repeated three times. The fungal strains IS2, IS5, IS7, IS8, C1, EC77 and EC82 were isolated from cranberry, B1 from blueberry, R1 from rose flower petals, F1, F4 and F5 from strawberry, T1 and T2 from tomato, VD1, VD2, FG5 and FG6 from olive tree; oomycetes PI3 and PI4 were isolated from olive tree. For details on strains, see Supplementary Table 1. The bars represent the standard error. Dots are individual data points. The growth medium for *Phytophthora infestans* (PI3 and PI4) and *Verticillium dahliae* (VD1 and VD2) was V8, whereas all other fungi were grown on yeast-glycerol agar plates.

EC4 was able to slow down the growth of all the above microbes by 13 to 70%, with the notable exception of *Penicillium* sp. strain IS8 (Figure 1 and Supplementary Tables 1, 2). The strongest growth suppression was noted for the two fungi, *Botrytis cinerea* strain R1 and *Diaporthe vaccinii* strain IS7. *B. cinerea* is a necrotrophic fungus that causes diseases in various plant species, including gray mold in rose plants (Ha et al., 2021) and yellow rot in cranberry fruit (Sabaratnam et al., 2016), while *D. vaccinii* is known to cause upright dieback of cranberry plant shoots and viscid rot as well as field and storage rot of the berries (Oudemans et al., 1998; Michalecka et al., 2017).

**FIGURE 2:**
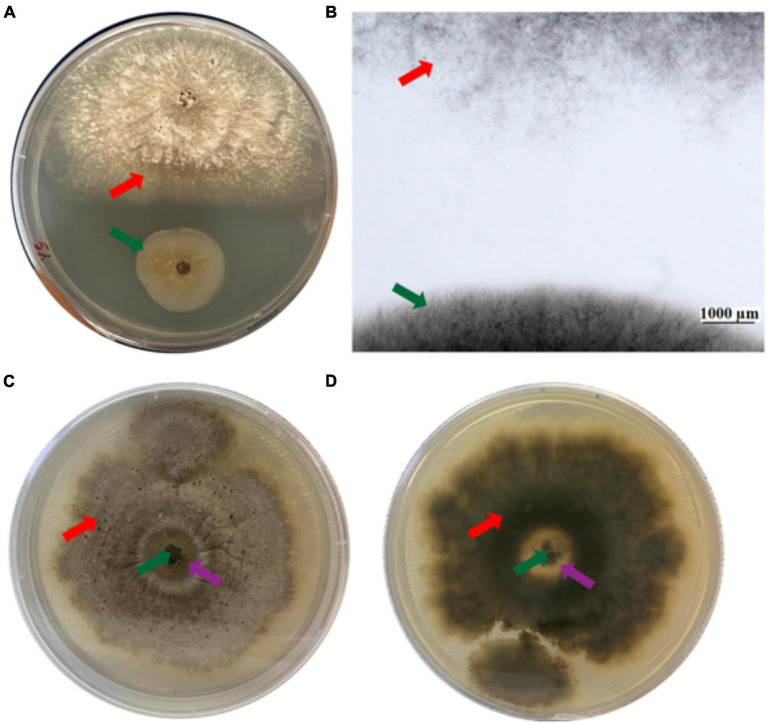
Inhibition of *Diaporthe* growth by EC4. The red arrow points to *Diaporthe* and the green arrow to EC4. **(A)** Dual-culture assay on an agar plate, top view. The tests were conducted in triplicates and repeated three times. **(B)** Stereo microscopic image of the inhibition zone observed in the dual culture assay. **(C,D)** A plate colonized by *Diaporthe* was inoculated with EC4, showing partial clearance of *Diaporthe* 21 days after inoculation. The purple arrows point to the partial clearance zone. **(C)** Top view and **(D)** bottom view of the plate.

The effect of EC4 on *D. vaccinii* was examined in more detail because it was originally isolated from diseased cranberry and is one of the important pathogens to impact cranberry crop. Ten days after simultaneous inoculation of EC4 and *Diaporthe* on an agar plate, a growth inhibition zone of *Diaporthe* became clearly visible to the eye (Figure 2A), and microscopic inspection of this zone revealed the absence of any fungal hyphae (Figure 2B). We also tested if EC4 can slow down the growth of *Diaporthe* on a petri dish already colonized by the pathogen. Indeed, 3 weeks after the inoculation with EC4, a clearance zone appeared and expanded in *Diaporthe*’s tuft (Figures 2C, D). In this zone, the pathogen’s mycelia did not completely disappear but were reduced considerably.

### 3.2 *In planta* pathogenicity test of presumed pathogens isolated from cranberry plants

The microbial strains tested in this study are considered pathogenic because ribotyping assigned them to species that have been experimentally demonstrated to be plant pathogens, yet experimental data are unavailable for the particular strains. Therefore, we infected aseptically grown cranberry plantlets with either of the seven microbes isolated from cranberry plants (C1, EC77, EC82, IS2, IS5, IS7, and IS8) and monitored plant health. Plantlets inoculated with *D. vaccinii* (IS7) and *Colletotrichum* sp. (EC77) died within 40 days (Supplementary Figure 1), but all other tested alleged pathogens showed no phenotypical effect on their host plant. A possible explanation for this observation is that the latter five microbes are latent pathogens under the tested conditions or, alternatively, only attack the berries but not the other plant parts.

### 3.3 The cranberry-plant pathogen *Diaporthe vaccinii* can be controlled by EC4

**FIGURE 3:**
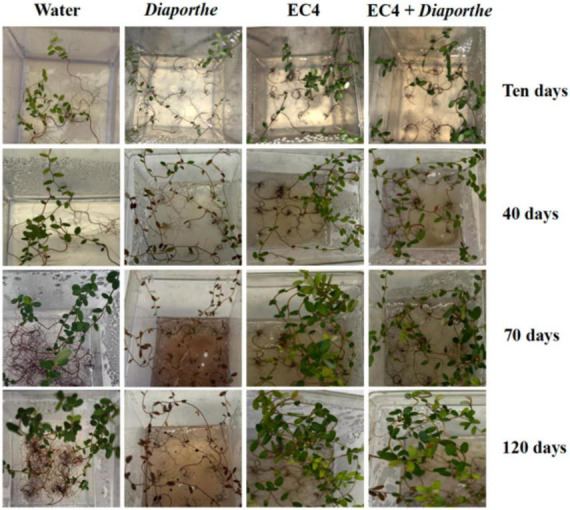
*In planta* pathogenicity test of *Diaporthe* and biocontrol test of EC4. Experiments were performed in triplicates and repeated three times for each condition. Cranberry plants were inoculated with sterile distilled water as a control, with *Diaporthe* alone, EC4 alone, or both, and inspected after 10, 40, 70, and 120 days. After 10 days, the health of *Diaporthe*-inoculated plants is visibly affected, and they died after 40 days. Plants inoculated with both *Diaporthe* and EC4 remained vigorous throughout the 3 months.

We examined whether EC4 is able to suppress *Diaporthe in planta*. For that, leaves of endophyte-free plantlets were inoculated with EC4 alone, *Diaporthe* alone, and EC4 plus *Diaporthe* (Figure 3). Controls and EC4-inoculated plantlets grew well over the monitored period of 3 months, while those inoculated with *Diaporthe* started dying off 10 days after inoculation. However, plantlets inoculated simultaneously with EC4 and *Diaporthe* remained healthy throughout 120 days (and even longer, not shown), demonstrating the biocontrol ability of EC4. Biocontrol of other *Diaporthe* species (*D. longicolla*, *D. fukushii*, and *D. foeniculina*) has been described before (Divilov and Walker, 2016; Geiger et al., 2022), but not of *D. vaccinii*. Thus, EC4 appears to be the first described microbe able to suppress *D. vaccinii in planta*.

### 3.4 *D. vaccinii* triggers the secretion of antimicrobial compounds by EC4

**FIGURE 4:**
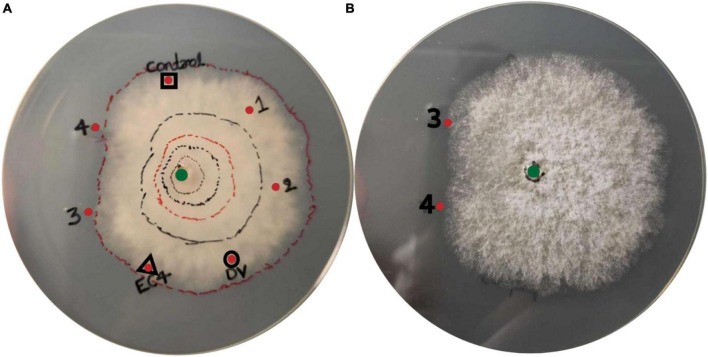
Presence of antimicrobial compounds in the EC4-*Diaporthe* co-culture medium. Dv, *D. vaccinii*. **(A)** Bottom view and **(B)** top view of the agar plate. *Diaporthe* has been inoculated at the center of the plate (green dot). Liquid medium from several cultures was tested for growth inhibition of *D. vaccinii*: a culture of EC4 alone (red dot framed by a triangle), *Diaporthe* alone (red dot framed by a circle), and a co-culture of EC4 and *Diaporthe* grown for 1–4 days (red dots labeled 1–4). Culture medium (2 ml) was concentrated 20× and placed 3 cm from the plate’s center. Sterile medium served as a control (red dot framed by a square). The growth of *Diaporthe* at room temperature was monitored for 15 days. Lines were drawn around the colony’s perimeter to document the growth in time. A conspicuous slow-down of radial growth occurred at dots 3 and 4. The tests were conducted in triplicates and repeated three times.

To investigate if EC4 produced antimicrobial compounds that suppress *Diaporthe*, we took aliquots of three different liquid media, notably from cultures in which either *Diaporthe* has grown alone, or EC4 has grown alone, or both have grown together, and tested if these aliquots confer growth inhibition when added to an agar plate inoculated with *Diaporthe*. The co-culture medium, but not that from EC4 alone or *Diaporthe* alone, caused an asymmetric radial growth of the pathogen (Figures 4A, B). This indicates that EC4 secretes antimicrobial compounds when in contact with *Diaporthe* but not in the absence of the pathogen.

### 3.5 The EC4 nuclear genome contains homologs of genes encoding hydrolytic enzymes

One of the mechanisms through which fungi suppress the growth of other fungi is by secreting cell-wall degrading enzymes (Jadhav et al., 2017). The targeted compounds are chitin, a β-(1,4)-linked *N*-acetylglucosamine polymer and β-(1,3)-glucan, galactomannans, α-glucans, and other carbohydrate polymers (Latgé, 2007). The nuclear genome of EC4 (Thimmappa et al., 2023) contains counterparts of 953 genes encoding Carbohydrate-Active enZymes (CAZymes) (Supplementary File 1). We predicted which of these enzymes remain inside the cell to serve the formation and maintenance of its own cell wall and which are likely secreted outside the cell and thus may degrade the cell wall of competing fungi. The CAZymes predicted to be secreted comprise between 20 and 66 homologs each of chitin, glucan, and mannan-degrading enzymes (Table 1 and Supplementary File 1). Further, EC4 encodes 46 likely secreted glucose–methanol–choline (GMC) oxidoreductases (Table 1 and Supplementary File 1), known to be involved in the biocontrol of fungi by fungi (Kawabe et al., 2011).

**TABLE 1:** EC4 genes predicted to code for enzymes degrading fungal and oomycete cell walls.

Targeted cell wall component	Nr. of genes	Nr. of expressed genes observed^a^	Nr. of genes with secretory signal	Nr. of expressed genes with secretory signal	Differentially upregulated genes of EC4 when grown together with *Diaporthe*
Chitin degrading enzymes	122	79	66	48	16
Glucan degrading enzymes	143	110	61	44	17
Mannan degrading enzymes	81	70	20	18	7
Cellulose degrading enzymes	59	44	42	33	2
Glucose–methanol–choline (GMC) oxidoreductase	46	35	23	18	2
Total count	451	338	212	191	44

^a^Expression data refer to four conditions: EC4 cultured in standard medium, in standard medium supplemented with cranberry plant extract [not shown], in the presence of *Diaporthe*, in the presence of live cranberry plant roots (Thimmappa et al., 2023). TPM > 0.1 is considered expressed.

As mentioned above, EC4 inhibited the growth of the oomycete *Phytophthora*, the cell wall of which contains cellulose instead of chitin (Thines, 2018). Indeed, the EC4 genome encodes 42 homologs of cellulase enzymes predicted to be secreted (Table 1 and Supplementary File 1). Such genes are also present in the genomes of fungi and bacteria, for which the ability to suppress *Phytophthora* has been demonstrated experimentally (Admassie et al., 2022; Frascella et al., 2022).

### 3.6 The EC4 nuclear genome encodes genes predicted to participate in secondary metabolite synthesis

Another important biocontrol mechanism, besides cell-wall hydrolysis, is the production of secondary metabolites. Among the well-known compound classes are nonribosomal peptides, polyketides, hybrid nonribosomal peptides/polyketides, terpenes, and indoles, many of which act as toxins, antibiotics, or cytostatics. Secondary metabolites are assembled by specialized enzymes, the genes of which are organized in biosynthetic gene clusters, together with genes encoding tailoring enzymes that further modify the molecules, and genes involved in regulation and transport (Medema et al., 2011). The EC4 nuclear genome contains 58 putative secondary metabolite gene clusters, about half of which are type-1 polyketide synthase (PKS) clusters, nonribosomal peptide synthetase (NRPS) clusters and hybrid clusters (Table 2 and Supplementary Table 3). Nineteen of these share substantial similarities with clusters from other organisms and for which the exact product is known, whereas no information is available for the compounds synthesized by the remaining 39 clusters.

**TABLE 2:** Putative secondary metabolite gene clusters in the EC4 genome.

Number	Gene cluster type	Transport-related genes	Cluster expressed^a^	Product and biological control principles of most similar known cluster	Differentially upregulated cluster of EC4 when grown with a pathogen	Similarity^b^
1	T1PKS	Major facilitator transporter, ABC transporter	Yes		No	
2	T1PKS	Sugar transport protein, drug resistance transporter	Yes		No	
3	NRPS–T1PKS	Drug resistance transporter	Yes		No	
4	T1PKS		Yes		No	
5	Indole		No		No	
6	T1PKS–NRPS	Drug resistance transporter	Yes	Wortmanamide A/B	Yes	66%
7	Terpene		Yes		No	
8	T1PKS	Drug resistance transporter, major facilitator transporter	Yes		Yes	
9	T1PKS	ABC transporter	Yes		No	
10	NRPS	Sugar transport protein	Yes		No	
11	Indole		Yes		No	
12	NRPS	ABC transporter, major facilitator transporter	Yes	Communesin A/B/C/D/E/G/H	Yes	25%
13	T3PKS	Sugar transport protein	Yes		No	
14	NRPS	ABC transporter	Yes	Oxaleimide C	Yes	10%
15	NRPS–T1PKS	ABC transporter, MATE efflux family protein	Yes	Dimethylcoprogen	No	100%
16	NRPS	ABC transporter, drug resistance transporter	No		No	
17	T1PKS	Drug resistance transporter	Yes		No	
18	NRPS	EamA family transporter, ABC transporter	Yes		No	
19	T1PKS	Sugar transport protein, Drug resistance transporter	Yes		No	
20	T1PKS		Yes		No	
21	NRPS	Drug resistance transporter	No		No	
22	NRPS	Drug resistance transporter, major facilitator transporter, sugar transport protein, ABC transporter	No		No	
23	Terpene		Yes		No	
24	T1PKS	Major facilitator transporter	Yes	Nectriapyrone C/D/nectriapyrone	No	100%
25	Terpene		Yes		No	
26	T1PKS	Sugar transport protein, drug resistance transporter	No	4-epi-15-epi-brefeldin A	No	20%
27	NRPS		Yes		Yes	
28	NRPS–T1PKS	Major facilitator transporter	Yes	Chaetoglobosin P/K/A	Yes	16%
29	NRPS	Drug resistance transporter	No		No	
30	T1PKS	Sugar transport protein, major facilitator transporter	No		No	
31	T1PKS–indole		Yes	Ankaflavin/monascin/ rubropunctatin/monascorubrin	No	8%
32	T1PKS	ABC transporter, drug resistance transporter	Yes		No	
33	T1PKS		Yes	Scytalone/T3HN	No	40%
34	T1PKS		Yes	Betaenone A/B/C	No	50%
35	NRPS	ABC transporter, major facilitator transporter	No		No	
36	Indole	Drug resistance transporter	No		No	
37	T1PKS	Drug resistance transporter, major facilitator transporter	Yes		No	
38	T1PKS	Sugar transport protein	Yes		No	
39	NRPS	ABC transporter	Yes		Yes	
40	Indole		Yes		No	
41	T1PKS	Drug resistance transporter	Yes	Fusarin C	No	100%
42	NRPS–T1PKS	Drug resistance transporter	No		No	
43	T1PKS	Sugar transport protein	Yes	(-)-Mellein	Yes	100%
44	NRPS-terpene		Yes	Sansalvamide	Yes	40%
45	NRPS		No		No	
46	Indole	Sugar transport protein	Yes		No	
47	T1PKS	Drug resistance transporter	No		No	
48	T1PKS–fungal-RiPP	Major facilitator transporter, drug resistance transporter	Yes	Neosartorin	No	21%
49	T1PKS		No	Burnettiene A/preburnettiene B/A	No	25%
50	T1PKS	Drug resistance transporter	Yes	Squalestatin S1	Yes	9%
51	NRPS		Yes		No	
52	T1PKS	Drug resistance transporter	No		No	
53	Indole	Major facilitator transporter	No		No	
54	T1PKS	Drug resistance transporter	Yes	Neosartorin	No	73%
55	NRPS–T1PKS	ABC transporter	No	Trichobrasilenol/xylarenic acid B/brasilane A/F/E/D	No	40%
56	T1PKS	ABC transporter	Yes		No	
57	NRPS		Yes		No	
58	Terpene		Yes	Squalestatin S1	No	40%
Total count			42 expressed, 15 not expressed		10 upregulated	

^a^Expression data refer to four conditions: EC4 cultured in standard medium, in standard medium supplemented with cranberry plant extract [not shown], in the presence of *Diaporthe*, in the presence of live cranberry plant roots (Thimmappa et al., 2023). TPM > 0.1 is considered expressed.

^b^Similarity was measured using the ClusterCompare algorithm of the antiSMASH tool; for details, refer to Blin et al., 2021.

### 3.7 Gene expression of EC4 when co-cultured with the pathogen

Of the 17,582 protein-coding genes encoded by the EC4 nuclear genome, about 89% are transcriptionally expressed under four conditions tested (Supplementary File 2), suggesting that essentially all genes are expressed under one or more of the various metabolic, physiological, and environmental conditions the fungus encounters during its life. To get insight into the genes involved in biocontrol, we investigated which EC4 genes change their expression when the organism is co-cultured with *Diaporthe*.

Table 3 compiles the transcriptional behavior of EC4 genes in the presence of the pathogen, with a focus on the genes coding for secondary metabolites and secreted hydrolytic enzymes. We refer to this subset of EC4 genes as “*Diaporthe*-growth-quelling candidates” or short “quelling candidates.” About ∼11% of these genes were upregulated when EC4 was cultured in the presence of the pathogen (for details, see Supplementary File 2). More specifically, the upregulated quelling candidates are predicted to encode 16 chitinases, 17 glucanases, and seven mannanases (Table 1, and Supplementary File 1) that were demonstrated in the biocontrol agent *Trichoderma* to be involved in inhibiting the growth of phytopathogens (Lopes et al., 2012; Geraldine et al., 2013; Vos et al., 2015). Of the ten differentially upregulated biosynthetic gene clusters, seven are predicted to produce antifungal compounds such as (-)-Mellein, Chaetoglobosin, and Oxaleimide (Chen et al., 2015; Perlatti et al., 2020; Reveglia et al., 2020), and putative cytotoxic compounds such as Sansalvamide, Communesin, Squalestatin S1, and Wortmanamide (see Table 2; Belofsky et al., 1999; Trost and Osipov, 2015; Bonsch et al., 2016; Hai and Tang, 2018). The compounds synthesized by the remaining three clusters are unknown but are assumed to act as antifungals as well.

**TABLE 3:** Transcript-level expression of EC4 genes in the presence of *Diaporthe*.

Nr. of protein-coding genes in EC4	17,582
Nr. of protein-coding EC4 genes with isoforms	3,492
Nr. of isoforms	10,984
Nr. of genes with isoforms differing in their coding regions	2,787
Nr. of genes with isoforms differing in their 5′UTRs	2,489
Nr. of genes with isoforms differing in their 3′UTRs	3,454
Nr. of upregulated genes in the presence of *Diaporthe*	1,809
Nr. of upregulated transcripts in the presence of *Diaporthe*	2,094
Nr. of downregulated genes in the presence of *Diaporthe*	1,341
Nr. of downregulated transcripts in the presence of *Diaporthe*	1,493
Nr. of genes with unchanged expression in the presence of *Diaporthe*	11,401
Nr. of transcripts with unchanged expression in the presence of *Diaporthe*	16,422
Nr. of biocontrol genes	509
Portion of expressed biocontrol genes^a^	75%
Portion of biocontrol genes differentially upregulated in the presence of *Diaporthe*	11%

^a^Transcript expression data refer to four conditions: EC4 cultured in standard medium, in standard medium supplemented with cranberry plant extract [not shown], in the presence of *Diaporthe*, and in the presence of live cranberry plant roots (Thimmappa et al., 2023). TPM > 0.1 is considered expressed.

### 3.8 The role of mRNA splicing isoforms in EC4

Previous transcriptome analysis showed that of the 17,582 EC4 nuclear genes for which we detected transcripts, nearly 20% produce mRNA splicing isoforms (Thimmappa et al., 2023). Here, we analyzed the types of alternative splicing and the function of isoforms, first globally and then with a focus on *Diaporthe*-quelling candidates. Globally, five distinct modes of alternative splicing were observed in EC4: intron retention (the most frequent event), exon skipping, use of alternative 5′ and 3′ splice sites, and alternative use of last exon (Table 4 and Supplementary File 3). Mutually exclusive exons and alternative first exon usage were not observed. Among the genes with isoforms, those differing in their coding regions amounted to 80%, while changes in their UTRs were observed in nearly all instances (Table 3). Distinct UTRs of alternative transcripts are thought to influence mRNA expression by changing the binding platform of regulatory microRNAs (Preussner et al., 2020). A similar pattern of alternative splicing, as seen in EC4, has been reported for other fungi (Fang et al., 2020; Ibrahim et al., 2021).

**TABLE 4:** Alternative splicing of nuclear EC4 genes.

Types of alternative splicing	Number
Intron retention	3,210
Alternative 3′ splice site	277
Alternative 5′ splice site	291
Exon skipping	21
Alternative first exon	8
Alternative last exon	0
Mutually exclusive exons	0

In EC4, 61 of the quelling candidates have isoforms, summing up to a total of 170. Of these isoforms, 16 (9%) were upregulated when EC4 was cultivated in the presence of *Diaporthe* (Supplementary File 3). The corresponding genes include chitinase, GMC oxidoreductase and others. Interestingly, alternative splicing events of 34 quelling candidates resulted in a change of conserved protein domains or targeting signals, changes that are bound to entail functional consequences for the translation product (Supplementary File 3).

**FIGURE 5:**
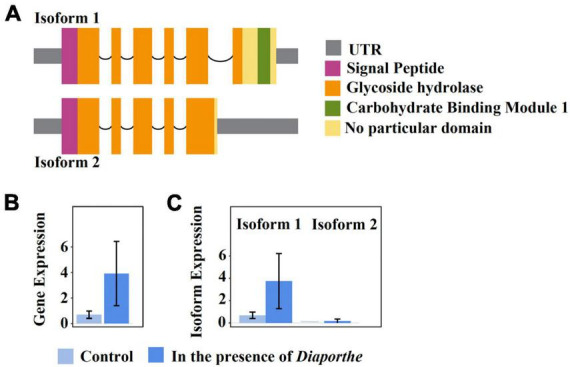
The isoform expression of the EC4 gene FUNEC_09200 gene when the organism was cultured alone or together with *D. vaccinii*. Isoform 1 is differentially upregulated in the presence of the pathogen. **(A)** Domain content of the two transcript isoforms. Boxes indicate UTRs and exons, and arcs symbolize introns. Protein domains were predicted using Pfam (Mistry et al., 2021). **(B,C)**, light blue and darker blue boxes represent transcriptional gene expression when EC4 was cultured alone or together with *D. vaccinii*, respectively. **(B)** Cumulative expression of all transcripts of the gene. **(C)** Expression of individual isoforms. Error bars indicate a 95% confidence interval. Plots were created using IsoformSwitchAnalyzeR v2.1.2 (Vitting-Seerup and Sandelin, 2019).

One remarkable example of a quelling-candidate gene with isoforms encoding different protein domains is FUNEC_09200 (Figure 5A). The protein product was annotated by our pipeline as a chitinase that is secreted outside the cell; secretion was corroborated by an additional InterPro scan analysis (Quevillon et al., 2005). Both isoforms are transcriptionally expressed in the absence as well as in the presence of *Diaporthe* (Figure 5B), but only isoform 1 is differentially upregulated under the latter condition (Figure 5C). While both isoforms comprise a chitinase domain belonging to the GH18 family of glycoside hydrolases (Cantarel et al., 2009), isoform 1 carries, in addition, a family-1 carbohydrate-binding module (CBM), which is non-catalytic. Members of this latter family bind cellulose, and some were shown to bind chitin as well (Linder et al., 1996). CBMs present in CAZymes were demonstrated to enhance the cell-wall degrading activity of the enzyme’s catalytic domain by binding to polymers that are in close proximity to the cognate substrate of the catalytic domain (Hervé et al., 2010). Further, in *Trichoderma reesei*, a family-1 CBM (designated Cel7A), when fused by genetic engineering to a chitinase-only enzyme, strongly improved the degradation of insoluble chitin (Neeraja et al., 2010; Kowsari et al., 2014). This indicates that co-cultivation with *Diaporthe* triggers differential up-regulation of isoform 1, augmenting the efficiency of a secreted EC4 chitinase that most likely participates in growth inhibition of the pathogen.

The question arises how EC4 protects itself against the chitinases it secreted into the culture medium. One possibility is that EC4 camouflages its exposed chitin molecules by secreting proteins exclusively consisting of CBMs with a high affinity to chitin. The EC4 genome encodes a number of genes that could serve this purpose. The mechanism of fungal camouflage is well described in pathogenic model fungi as a strategy employed during the colonization of plants to interfere with the activation of chitin-triggered plant immunity (Pusztahelyi, 2018). It would be interesting to examine by proteomics, metabolomics and enzymatics the molecular underpinning of EC4’s chitinase-mediated suppression of *Diaporthe* and the means of self-protection against these potent antifungal enzymes.

## 4 Conclusion and outlook

EC4, an endosymbiont of *Vaccinium macrocarpon*, is one of the few fungal endophytes from the chaetosphaeriaceae family uniting the capacities to promote plant growth (Thimmappa et al., 2023) and to suppress plant pathogens. EC4 protects cranberry plantlets from *Diaporthe* infection and, in addition, inhibits various other pathogens from taxonomically diverse host plants. Furthermore, when in contact with *Diaporthe vaccinii*, EC4 expresses hydrolytic enzymes and secondary metabolites recognized for their roles in biocontrol. The described variation in transcription behavior when in contact with this pathogen makes EC4 a prime organism for the investigation of the molecular mechanisms involved in dynamic interactions between microbe species sharing the same plant host as their habitat. At the same time, EC4 is a promising candidate for large-scale sustainable application in industrial cranberry farming, particularly in North America, where it is an economically important crop plant.

## Data availability statement

The datasets presented in this study can be found in online repositories. The names of the repository/repositories and accession number(s) can be found in this article/Supplementary material.

## Author contributions

BCT: Investigation, Writing – original draft. LS: Investigation, Writing – review & editing. LF: Investigation, Writing – review & editing. MS: Investigation, Writing – review & editing. PB: Investigation, Writing – review & editing. BH: Investigation, Writing – review & editing. BFL: Conceptualization, Funding acquisition, Writing – review & editing. GB: Conceptualization, Funding acquisition, Supervision, Writing – review & editing.

## Footnotes

1
http://www.ncbi.nlm.nih.gov/blast/

2
www.cazy.org

## Supplementary material

Supplementary File 1 CAZymes.

Supplementary File 2 CAZymes.

Supplementary File 3 CAZymes.
