# Supplementary material for: A biofertilizing fungal endophyte of cranberry plants suppresses the plant pathogen Diaporthe
Source: Front Microbiol. 2024 Feb 2;15:1327392. doi: 10.3389/fmicb.2024.1327392 (PMC10869595; doi:10.3389/fmicb.2024.1327392)
Supplement: Supplementary file 4 [file Data_Sheet_1.PDF]

A biofertilizing fungal endophyte of cranberry plants suppresses the plant pathogen *Diaporthe*

Bhagya C. Thimmappa, Lila Naouelle Salhi, Lise Forget, Matt Sarrasin, Peniel Bustamante Villalobos, Bernard Henrissat, B. Franz Lang and Gertraud Burger

Table S1. Microbial strains used in this study

Kingdom	Strains	Species	Host plant	Organismal description/reference	Accession number	Source
Fungi	EC4	*Codinaeella* sp.	Cranberry	Cranberry plant growth promoting endosymbiont (Thimmappa et al., 2023).	OQ745738	In-house collection
	IS2	*Alternaria alternata*	Cranberry	Cranberry fruit rot pathogen (Olatinwo et al., 2003).	ON350796	R. Belanger, Université Laval, QC, Canada
	IS5	*Peniophora* sp.	Cranberry	Root canker of apples (Taylor, 1969).	ON350797	
	IS7	*Diaporthe vaccinii*	Cranberry	Upright dieback of cranberry plant shoots and viscid rot of the cranberries (Michalecka et al., 2017).	ON350798	
	IS8	*Penicillium* sp.	Cranberry	Cranberry fruit rot pathogen (Jeffers, 1991).	ON350799	
	C1	*Physalospora vaccinii*	Cranberry	Blotch rot of cranberries (Conti et al., 2019).	ON350807	Current work
	EC77	*Colletotrichum* sp.	Cranberry	Bitter rot of cranberries (Waller et al., 2020).	OL342684	
	EC82	*Godronia* sp.	Cranberry	Cranberry fruit rot pathogen (Conti et al., 2020).	OL342688	
	B1	*Alternaria alternata*	Blueberry	Blueberry fruit rot pathogen (Zhu and Xiao, 2015) and cranberry fruit rot pathogen (Olatinwo et al., 2003).	ON350803	
	F1	*Rhizopus stolonifer*	Strawberry	Rhizopus rot of strawberry (Lin et al., 2017), cranberry fruit rot pathogen (Blodgett et al., 2002).	ON350800	
	F4	*Cadophora luteo-olivacea*	Strawberry	Petri disease and esca of grapevine (Maldonado-González et al., 2020).	ON350802	
	F5	*Botrytis cinerea*	Strawberry	A gray mold of strawberries (Petrasch et al., 2019) and yellow rot in cranberry fruit (Olatinwo et al., 2004).	NA	
	T1	*Alternaria alternata*	Tomato	Early blight in tomato 12/28/23 6:03:00 PM and cranberry fruit rot pathogen (Olatinwo et al., 2004).	ON350808	
	T2	*Alternaria alternata*	Tomato	Early blight in tomato 12/28/23 6:03:00 PM and cranberry fruit rot pathogen (Olatinwo et al., 2004).	ON350809	
	R1	*Botrytis cinerea*	Rose flower	Grey mold in rose plants (Ha et al., 2021) and yellow rot in cranberry fruit (Sabaratnam et al., 2016).	ON350806	
	FG5	*Fusarium graminearum*	Olive Tree	Olive tree root rot and dieback (Chliyeh et al., 2017), and	NA	K. Aliferis (Agricultural University of Athens, Greece)
	FG6	*Fusarium graminearum*	Olive Tree	cranberry fruit rot pathogen (Sabaratnam et al., 2016).	NA	
	VD1	*Verticillium dahlia*	Olive Tree	Verticillium wilt of olive trees (Montes-Osuna and	NA	
	VD2	*Verticillium dahlia*	Olive Tree	Mercado-Blanco, 2020).	NA	
Oomycete	PI3	*Phytophthora infestans*	Olive Tree	Causes root and collar rot in olive trees (Cacciola et al.,	NA	
	PI4	*Phytophthora infestans*	Olive Tree	2001) and root rot in cranberry plants (Oudemans, 1999).	NA	

NA – Not Available

Table S2. Growth inhibition of plant pathogens by EC4

Kingdom	Isolate	Species	Growth inhibition (%)	chi-squared[a]	p-value[a]	Growth Medium
Fungi	IS7	*Diaporthe vaccinii*	68.1	5.67	0.01725	Yeast-glycerol agar
	C1	*Physalospora vaccinii*	48.1	6.14	0.01324	
	IS2	*Alternaria alternata*	47.5	6.05	0.01387	
	EC77	*Colletotrichum gloeosporioides*	39.4	5.67	0.01725	
	EC82	*Godronia cassandrae*	30.6	6.14	0.01324	
	IS5	*Peniophora* sp.	23.8	6.14	0.01324	
	IS8	*Penicillium* sp.	0	/	/	
	R1	*Botrytis cinerea*	70.0	4.50	0.03389	
	F4	*Cadophora luteo-olivacea*	60.0	5.00	0.02535	
	FG5	*Fusarium graminearum*	53.0	4.50	0.03389	
	FG6	*Fusarium graminearum*	50.0	4.50	0.03389	
	T1	*Alternaria alternata*	40.0	4.50	0.03389	
	F5	*Botrytis cinerea*	39.0	4.50	0.03389	
	T2	*Alternaria alternata*	36.0	4.30	0.0369	
	B1	*Alternaria alternata*	32.0	4.30	0.0369	
	F1	*Rhizopus* sp.	13.0	5.00	0.02535	
Fungi	VD1	*Verticillium dahlia*	26.0	4.50	0.03389	V8 agar medium
	VD2	*Verticillium dahlia*	21.0	4.50	0.03389	
Oomycetes	PI3	*Phytophthora infestans*	44.0	4.50	0.03389	
	PI4	*Phytophthora infestans*	38.0	4.50	0.03389	

[a] Determined by the nonparametric Kruskal–Wallis test (Ogle et al., 2023).

Table S3. Putative secondary metabolite gene clusters in the EC4 genome

#	Contig and Region	Gene cluster type	From	To	ID of the biosynthetic gene in the cluster	Gene expressed[a]	Transport-related genes in the cluster	Product of most similar known cluster	Differentially expressed clusters, when grown with a pathogen	Similarity[b]
1	NODE_2 (Region 2.1)	T1PKS	317,693	385,400	FUNEC_12092	Yes	FUNEC_12194 and FUNEC_12353 - Major facilitator transporter, FUNEC_12399 - ABC transporter	/	No	/
2	NODE_3 (Region 3.1)	T1PKS	787,770	855,538	FUNEC_04276	Yes	FUNEC_04459 - Sugar transport protein, FUNEC_04235 and FUNEC_04395 - Drug resistance transporter	/	No	/
3	NODE_4 (Region 4.1)	NRPS - T1PKS	6,996	91,989	FUNEC_05999, FUNEC_05981	Yes	FUNEC_06037 - Drug resistance transporter	/	No	/
4	NODE_4 (Region 4.2)	T1PKS	332,260	395,122	FUNEC_05774	Yes	/	/	No	/
5	NODE_4 (Region 4.3)	indole	691,095	722,321	FUNEC_05969	No	/	/	No	/
6	NODE_4 (Region 4.4)	T1PKS - NRPS	793,100	865,977	FUNEC_05809	Yes	FUNEC_05928 - Drug resistance transporter	Wortmanamide A/B	Yes	66%
7	NODE_5 (Region 5.1)	terpene	549,900	581,495	FUNEC_08015	Yes	/	/	No	/
8	NODE_6 (Region 6.1)	T1PKS	716,575	785,076	FUNEC_04968	Yes	FUNEC_04680 - Drug resistance transporter, FUNEC_04828 - Major facilitator transporter	/	Yes	/
9	NODE_8 (Region 8.1)	T1PKS	140,682	207,990	FUNEC_07067, FUNEC_06968	Yes	FUNEC_06932 and FUNEC_07089 - ABC transporter	/	No	/
10	NODE_9 (Region 9.1)	NRPS	734,915	785,236	FUNEC_13856	Yes	FUNEC_13743 - Sugar transport protein	/	No	/
11	NODE_10 (Region 10.1)	Indole	101,887	133,290	FUNEC_15749	Yes	/	/	No	/
12	NODE_12 (Region 12.1)	NRPS	6,298	89,664	FUNEC_02287	Yes	FUNEC_02270 - ABC transporter, FUNEC_02135 - Major facilitator transporter	Communesin A/ B/ C/ D/E/ G/ H	Yes	25%
13	NODE_12 (Region 12.2)	T3PKS	426,147	487,388	FUNEC_02247	Yes	FUNEC_02202 and FUNEC_02204 - Sugar transport protein	/	No	/
14	NODE_16 (Region 16.1)	NRPS	9,804	103,227	FUNEC_10438	Yes	FUNEC_10430 - ABC transporter	Oxaleimide C	Yes	10%
15	NODE_16 (Region 16.2)	NRPS - T1PKS	378,497	512,529	FUNEC_10371	Yes	FUNEC_10515 - ABC transporter, FUNEC_10517 - MATE efflux family protein	Dimethylcoprogen	No	100%
16	NODE_18 (Region 18.1)	NRPS	29,443	92,994	FUNEC_01366	No	FUNEC_01361 - ABC transporter, FUNEC_01280 - Drug resistance transporter	/	No	/
17	NODE_18 (Region 18.2)	T1PKS	370,765	436,669	FUNEC_01328	Yes	FUNEC_01251 - Drug resistance transporter	/	No	/
18	NODE_24 (Region 24.1)	NRPS	284,315	370,097	FUNEC_11852, FUNEC_11859	Yes	FUNEC_11834 - EamA family transporter, FUNEC_11884 - ABC transporter	/	No	/
19	NODE_25 (Region 25.1)	T1PKS	364,068	432,232	FUNEC_10305	Yes	FUNEC_10245 and FUNEC_10258 - Sugar transport protein, FUNEC_1032 - Drug resistance transporter	/	No	/
20	NODE_31 (Region 31.1)	T1PKS	15,739	84,381	FUNEC_03333	Yes	/	/	No	/
21	NODE_33 (Region 33.1)	NRPS	139,986	205,493	FUNEC_07234	No	FUNEC_07177 - Drug resistance transporter	/	No	/
22	NODE_34 (Region 34.1)	NRPS	334,314	398,084	FUNEC_13106	No	FUNEC_13091 - Drug resistance transporter,	/	No	/

							FUNEC_13055 - Major facilitator transporter, FUNEC_13026 - Sugar transport protein, FUNEC_13171 - ABC transporter			
23	NODE_36 (Region 36.1)	terpene	92,336	123,643	FUNEC_06627	Yes	/	/	No	/
24	NODE_38 (Region 38.1)	T1PKS	1,966	70,210	FUNEC_03172	Yes	FUNEC_03194 - Major facilitator transporter	Nectriapyrone C/ D/nectriapyrone	No	100%
25	NODE_38 (Region 38.2)	terpene	261,510	293,585	FUNEC_03192	Yes	/	/	No	/
26	NODE_41 (Region 41.1)	T1PKS	204,102	271,759	FUNEC_14612	No	FUNEC_14601 and FUNEC_14645 - Sugar transport protein, FUNEC_14558 - Drug resistance transporter	4-epi-15-epi-brefeldin A.	No	20%
27	NODE_42 (Region 42.1)	NRPS	1	63,900	FUNEC_01513	Yes	/	/	Yes	/
28	NODE_50 (Region 50.1)	NRPS - T1PKS	143,025	260,774	FUNEC_08884, FUNEC_08865	Yes	FUNEC_08898 - Major facilitator transporter	Chaetoglobosin P/ K/ A	Yes	16%
29	NODE_53 (Region 53.1)	NRPS	47,267	113,896	FUNEC_02911	No	FUNEC_02926 - Drug resistance transporter	/	No	/
30	NODE_55 (Region 55.1)	T1PKS	14,119	79,989	FUNEC_09777	No	FUNEC_09828 - Sugar transport protein, FUNEC_09768 and FUNEC_09800 - Major facilitator transporter	/	No	/
31	NODE_55 (Region 55.2)	T1PKS-indole	256,650	313,558	FUNEC_09791	Yes	/	Ankaflavin/monascin/ rubropunctatine/ monascorubrin	No	8%
32	NODE_58 (Region 58.1)	T1PKS	249,846	298,168	FUNEC_11557	Yes	FUNEC_11561 - ABC transporter, FUNEC_11548 - Drug resistance transporter	/	No	/
33	NODE_64 (Region 64.1)	T1PKS	88,120	154,801	FUNEC_06565	Yes	/	Scytalone/T3HN	No	40%
34	NODE_67 (Region 67.1)	T1PKS	49,061	118,071	FUNEC_11106	Yes	/	Betaenone A/B/C	No	50%
35	NODE_70 (Region 70.1)	NRPS	1	38,287	FUNEC_10917	No	FUNEC_10883 and FUNEC_10908 - ABC transporter, FUNEC_10939 - Major facilitator transporter	/	No	/
36	NODE_74 (Region 74.1)	indole	76,624	108,020	FUNEC_11415	No	FUNEC_11369 - Drug resistance transporter	/	No	/
37	NODE_78 (Region 78.1)	T1PKS	20,551	82,583	FUNEC_16853	Yes	FUNEC_16925 - Drug resistance transporter, FUNEC_16860 - Major facilitator transporter	/	No	/
38	NODE_78 (Region 78.2)	T1PKS	109,358	176,890	FUNEC_16923, FUNEC_16877	Yes	FUNEC_16916 - Sugar transport protein	/	No	/
39	NODE_82 (Region 82.1)	NRPS	48,904	131,010	FUNEC_12753	Yes	FUNEC_12703 - ABC transporter	/	Yes	/
40	NODE_83 (Region 83.1)	indole	160,366	191,826	FUNEC_03583	Yes	/	/	No	/
41	NODE_86 (Region 86.1)	T1PKS	45,913	82,031	FUNEC_10743	Yes	FUNEC_10719 and FUNEC_10733 - Drug resistance transporter	Fusarin C	No	100%
42	NODE_86 (Region 86.2)	NRPS - T1PKS	86,687	164,790	FUNEC_10722, FUNEC_10749	No	FUNEC_10737 and FUNEC_10730 - Drug resistance transporter	/	No	/
43	NODE_90 (Region 90.1)	T1PKS	1	44,883	FUNEC_00955	Yes	FUNEC_00927 - Sugar transport protein	(-)-Mellein	Yes	100%
44	NODE_92 (Region 92.1)	NRPS - terpene	48,484	132,777	FUNEC_08454, FUNEC_08478, FUNEC_08443	Yes	/	Sansalvamide	Yes	40%
45	NODE_97 (Region 97.1)	NRPS	10,237	75,159	FUNEC_11523	No	/	/	No	/
46	NODE_97 (Region 97.2)	indole	173,203	204,512	FUNEC_11514	Yes	FUNEC_11524 - Sugar transport protein	/	No	/
47	NODE_99 (Region 99.1)	T1PKS	119,144	182,567	FUNEC_03828	No	FUNEC_03835 - Drug resistance transporter	/	No	/

48	NODE_108 (Region 108.1)	T1PKS-fungal-RiPP	65,900	158,064	FUNEC_11698, FUNEC_11704, FUNEC_11679	Yes	FUNEC_11690 - Major facilitator transporter, FUNEC_11710 - Drug resistance transporter	Neosartorin	No	21%
49	NODE_119 (Region 119.1)	T1PKS	117,685	155,514	FUNEC_17565, FUNEC_17572	No	/	Burnettiene A/preburnettiene B/ A	No	25%
50	NODE_120 (Region 120.1)	T1PKS	1	45,870	FUNEC_12912	Yes	FUNEC_12898 - Drug resistance transporter	Squalestatin S1	Yes	9%
51	NODE_122 (Region 122.1)	NRPS	36,109	100,140	FUNEC_07868	Yes	/	/	No	/
52	NODE_125 (Region 125.1)	T1PKS	52,001	115,170	FUNEC_00873	No	FUNEC_00903 and FUNEC_00875 - Drug resistance transporter	/	No	/
53	NODE_128 (Region 128.1)	Indole	117,959	141,365	FUNEC_02057	No	FUNEC_02067 - Major facilitator transporter	/	No	/
54	NODE_147 (Region 147.1)	T1PKS	41,396	117,965	FUNEC_09448, FUNEC_09484	Yes	FUNEC_09459 - Drug resistance transporter	Neosartorin	No	73%
55	NODE_162 (Region 162.1)	NRPS - T1PKS	12,442	84,593	FUNEC_08781	No	FUNEC_08780 and FUNEC_08789 - ABC transporter	Trichobrasilenol/xylarenic acid B/brasilane A/ F/ E/D	No	40%
56	NODE_163 (Region 163.1)	T1PKS	19,225	86,968	FUNEC_14100	Yes	FUNEC_14103 - ABC transporter	/	No	/
57	NODE_199 (Region 199.1)	NRPS	7,150	52,133	FUNEC_02885	Yes	/	/	No	/
58	NODE_215 (Region 215.1)	terpene	1	25,860	FUNEC_00407	Yes	/	Squalestatin S1	No	40%
Total count						42 expressed, 15 not expressed			10 upregulated	

[a]Expression data refer to four conditions: EC4 cultured in standard medium, in standard medium supplemented with cranberry plant extract [not shown], in the presence of *Diaporthe*, in the presence of live cranberry plant roots (Thimmappa et al., 2023). TPM > 0.1 is considered expressed.

[b]Similarity was measured using the ClusterCompare algorithm of the antiSMASH tool; for details, refer to Blin et al., 2021.

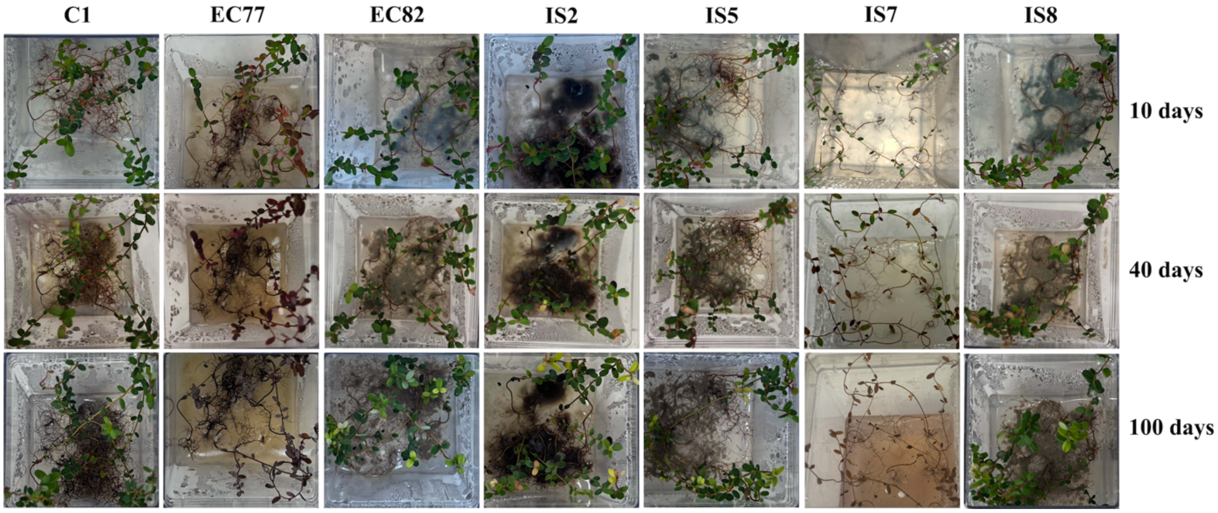

Figure S1. *In-planta* pathogenicity test of fungal strains isolated from diseased cranberry plants. Cranberry plantlets were inoculated with the presumed pathogens and inspected after 10, 40, and 100 days. C1 - *Physalospora vaccinii*, EC77 - *Colletotrichum* sp., EC82 - *Godronia* sp., IS2 - *Alternaria alternata*., IS5 - *Peniophora* sp., IS7 – *Diaporthe vaccinii*, IS8 - *Penicillium* sp.
